# Relationship between borderline personality features, emotion regulation, and non-suicidal self-injury in depressed adolescents: a cross-sectional study

**DOI:** 10.1186/s12888-023-04800-1

**Published:** 2023-04-28

**Authors:** Yang Chen, Wenxian Fu, Sifan Ji, Wei Zhang, Lingmin Sun, Tingting Yang, Kongliang He, Yongjie Zhou

**Affiliations:** 1 Affiliated Psychological Hospital of Anhui Medical University, Hefei, 230022 China; 2 Anhui Mental Health Center, Hefei, 230022 China; 3 School of Mental Health and Psychological Sciences, Anhui Medical University, Hefei, 230022 China; 4 Psychological counseling department, Hefei Fourth People’s Hospital, Anhui, 230000 China; 5 Shenzhen Mental Health Center, Shenzhen, Guangdong China

**Keywords:** Adolescents, Non-suicidal self-injury, Borderline personality features, Emotional regulation

## Abstract

**Objective:**

Non-suicidal self-injury (NSSI) is common among adolescents and has been linked to mental disorders and suicide in addition to physical injuries. According to the empirical avoidance model, adolescents with NSSI have stronger emotional affect and poorer emotional regulation than those without NSSI, and these constitute core features of borderline personality disorder (BPD). The relationship between borderline personality features, emotional regulation, and NSSI in the population is unclear. This study explored these associations to provide a theoretical basis for the treatment of NSSI in the future.

**Methods:**

Depressed adolescents (n = 1192) were evaluated using Chinese versions of the Function Assessment of Self-mutilation Scale, Emotional Regulation Questionnaire for Children and Adolescents, and Borderline Personality Features Scale for Children.

**Results:**

The majority of depressed adolescents (71.3%, 850/1192) had demonstrated NSSI in the past year, with cutting or scratching being the most common form (57.4%). Pearson correlation analysis with NSSI as a fixed factor (NSSI = 1, no NSSI = 2) revealed a negative correlation between NSSI and borderline personality features (r = -0.314, P < 0.01) but a positive correlation between NSSI and emotional regulation capacity (r = 0.159, P < 0.01), which was positively correlated with the expression suppression dimension (r = 0.079, p < 0.01); however, there was no significant correlation between the cognitive reappraisal dimension and expression suppression (r = 0.022, p > 0.05). The occurrence of NSSI was also positively correlated with borderline personality features in general (r = 0.314, p < 0.01). These results were statistically significant. Emotional regulation played a mediating role between borderline personality traits and NSSI in adolescents with depression (effect value = 0.151).

**Conclusion:**

Borderline personality features and emotional regulation ability were significantly correlated with NSSI in depressed adolescents. Borderline personality symptoms not only directly influenced NSSI risk in adolescents with depression, but also indirectly influenced NSSI risk through emotional regulation.

## Introduction

Non-suicidal self-injury (NSSI), defined as the deliberate destruction or alteration of body tissue without conscious suicidal intent, is increasingly recognized as a major public health concern. The concept and assessment of NSSI has also evolved in recent years, and it is listed as a distinct syndrome in the DSM-V “Conditions for Further Research“ [1]. This NSSI behavior most often occurs during adolescence [2] and has a high prevalence rate in both clinical and epidemiological samples. A recent longitudinal study of non-clinical adolescents reported a prevalence rate of over 17.2% [3]. Moreover, NSSI is a risk factor for both suicide and mental health problems such as depression and anxiety in the short-term [4–6], and a longer-term risk factor for more severe mental illness and suicide [7]. Therefore, identifying the risk factors for NSSI in adolescents will provide valuable clues to its etiology and contribute to the development of effective interventions.

According to the empirical avoidance model, adolescents exhibiting NSSI have stronger emotional affect, poorer emotional regulation, and a greater propensity for avoidance behaviors, particularly avoidance behaviors that are intended to relieve or distract from emotional pain (including the physical pain generated by NSSI ). Moreover, avoidant behaviors can increase over time [8, 9]. In contrast, other theoretical models posit interpersonal motivations for NSSI, such as changing the external environment and influencing others [10, 11]. For instance, Nock proposed a social theory in which NSSI is aimed at conveying emotional pain, eliciting caring and supportive responses, and strengthening relationships [12]. However, it is generally accepted that emotional regulation is the most common motivation underlying NSSI. Moreover, Turner and coworkers demonstrated that NSSI is associated with emotional regulation disorders [13].

Borderline personality disorder (BPD) is a severe mental disorder characterized by emotional instability, intense and volatile relationships, impulsivity, distorted or fragile self-image, paranoia, and frequent suicidal ideation [14]. The biosocial theory of BPD [15–17] proposes that emotional deficits, such as hypersensitivity, over-reactivity, a tendency for slow recovery from the physiological responses associated with emotions, and defects of emotional regulation represent core disease characteristics. While BPD is usually diagnosed in young adults, Crick et al. concluded that some adolescents exhibit relatively stable cognitive, emotional, and behavioral characteristics similar to those of adult BPD patients, and coined the term adolescent borderline personality features (BPFs) [18]. Subsequent studies on adolescent BPF found a higher risk of suicide, NSSI, and substance abuse as well as frequent risk-taking behaviors, seriously impaired social function, and poor overall mental health [19]. Given the high prevalence of BPF and its potentially devastating consequences on physical and mental health among adolescents, there is an urgent need to identify associated risk factors than can enhance prevention and early intervention efforts [20].

Previous studies have examined the associations between NSSI with BPFs and emotional regulation. However, NSSI is also frequently observed in depression, which is the most prevalent psychiatric disorder among adolescents globally and a major risk factor for suicidality. Depression and BPD can often co-occur. Up to 80% of people with BPD will experience one or more episodes of major depression in their lifetime, and up to half of people with BPD may experience persistent depressive symptoms [21]. The association between MDD and BPD is sometimes strong, and the clinical distinction between them is unclear, particularly in adolescents. Studies involving adults have shown significant overlap or co-occurrence between BPD and MDD, which may be influenced by different clinical phenotypes [22]. Therefore, we attempted to control for depressive symptoms to further elucidate the relationship between borderline personality traits and NSSI. Therefore, the current study examined the associations between BPF symptoms (emotional instability, identity disorder, poor family relations, negative emotional regulation, cognitive reappraisal, and expression suppression) and NSSI in adolescent patients with depression, and explored whether emotional regulation plays a mediating role between borderline personality traits and NSSI.

## Methods

### Participants

This cross-sectional study of NSSI in adolescents with depression was conducted from January to December 2021 at 12 hospitals across China. Questionnaires were prepared using the Situation×Trait Adaptive Response (STARx) Questionnaire, and 1192 valid questionnaires were collected, of which 877 were completed by females and 315 by males. All participants and guardians provided written informed consent.

### Inclusion and exclusion criteria

The inclusion criteria were as follows:

1. met the DSM-5 criteria defining a depressive episode or depressive episode in bipolar disorder (i.e., outpatient depression status met the criteria for a depressive episode);
2. 12 to 18 years old;
3.  ≥ 6 years of formal education;
4. no history of suicidal behavior;
5. right-handed;
6. patients and their families agreed to participate and provided written informed consent.

Candidates were excluded for :

1. severe physical, infectious, or immune system diseases;
2. traumatic brain injury, epilepsy, or other known severe neurological or organic brain disease;
3. history of severe mental disorders such as schizophrenia and intellectual disability.

### Measures

#### General demographic data

Sociodemographic information (age, sex, ethnicity) was collected using questionnaires designed by the research team.

#### Functional Assessment of Self-Mutilation

The Functional Assessment of Self-Mutilation (FASM) by Lloyd and coworkers is widely used for the assessment of adolescent NSSI [23]. This study used the C-FASM scale [24], which queries whether subjects have intentionally performed 10 specific self-injurious behaviors (such as cutting, burning, or intentionally beating oneself) within the past 12 months as well as the frequency, severity, duration, and method of such behaviors. Cronbach’s α for this study sample was 0.991.

#### Emotional regulation questionnaire for children and adolescents

The Emotional Regulation Questionnaire for Children and Adolescents (ERQ-CA) was developed by Gullone and Taffe [25] based on the emotional regulation process model and the adult version by Gross [26]. This study focused on two emotional regulation strategies commonly used by children and adolescents aged 10–18 years: cognitive reappraisal and expression suppression. Cognitive reappraisal is an emotional regulation strategy that reappraises emotional situations and thus changes emotional experience, while expression suppression refers to behaviors that suppress individual emotional experience. The ERQ-CA consists of 10 items, each rated using a five-point Likert scale (1 for “strongly disagree” to 5 for “strongly agree”). The cognitive reappraisal dimension subscale score ranges from 6 to 30 points, and the expression suppression dimension subscale score from 4 to 20 points, with higher scores indicating greater use of the corresponding strategy. In this study, Cronbach’s alpha was 0.726.

#### Borderline personality features scale for children

The Borderline Personality Features Scale for Children (BPFS-C) is a revised version for children and adolescents aged 9–18 years based on items of the Personality Assessment Inventory-Borderline Features Scale used for adults. The BPFS-C consists of items including affective instability (impulsivity, difficulty calming mood, more intense emotions), identity disorder (inconsistency of self-assessment, identity problems), negative relationships (emotional distress, feeling of isolation, negative evaluation of friends, paranoia), and self-harm (uncontrollable excessive behavior, lack of self-restraint). Each of these four dimensions is assessed by six items. Each item is scored according to a five-point Likert scale (1 = never, 2 = occasionally, 3 = sometimes, 4 = frequently, 5 = always) with four reverse scoring entries. Higher total scores indicate greater severity of BPFs. In this study, the Cronbach’s alpha was 0.870.

### Statistical analyses

All statistical analyses were conducted using SPSS v25.0 (IBM Corp., Armonk, NY, USA). Count data are expressed as [n] (%), and measurement data conforming to a normal distribution as mean ± standard deviation. Differences in demographic variables between adolescents with and without NSSI were evaluated by independent-samples t-test. Independent samples t-tests were also performed to evaluate differences in borderline personality features (cognitive reappraisal, expression inhibition, emotional instability, identity disorder, negative interpersonal relationship, and self-injury) between the NSSI group and non-NSSI group. Pearson’s correlation analysis was used to analyze the associations between BPFs, emotional regulation dimensions, and NSSI. For this analysis, NSSI was taken as a fixed factor (NSSI = 1, no NSSI = 2). For mediation analyses, BPFs (BPFC-S scores) were set as independent variables, and NSSI (Yes or NO) was taken as the dichotomous dependent variable. The mediating effect of the model was explored using the difference coefficient test.

## Results

### Demographics of the study group

A total of 1,192 patients from 12 hospitals across China were included in this study (average age 15.10 ± 1.63 years, 9.32 ± 1.74 years of formal education). Among the respondents, 1,091 were of Han ethnicity and 101 were from ethnic minorities. About 69.2% of the patients lived in urban areas, while the remainder lived in rural areas. The annual household income of the patients was 101,200 ± 24,400 Yuan. Among the 1,192 respondents, 850 reported NSSI in the past 12 months.

### Types and frequencies of NSSI

The types and frequencies of NSSI reported on the FASM by adolescents with depression are listed in Table 1. Of the ten NSSI behaviors considered, the most common type was deliberately cutting or scratching one’s skin, reported by more than half of the total number of respondents, followed by deliberately beating oneself, striking one’s fist or head against a hard object, and biting oneself deliberately (e.g., on the mouth or lips).

**Table 1:** Types and frequency of NSSI among adolescents with depression (n = 1192)

	Rate n (%)	Frequency of occurrence*
1. Deliberately cutting or scratching one’s skin	57.4	68.98 ± 276.62
2. Deliberately beating oneself	35.4	76.76 ± 176.28
3. Pulling his hair on purpose	23.8	96.51 ± 348.09
4. Deliberately etching words or designs on the body with sharp objects	19.8	44.66 ± 265.95
5. Deliberate irritation of the wound which interferes with healing	22.6	72.72 ± 268.74
6. Deliberately stabbing objects into the skin or nails	9.9	78.41 ± 374.48
7. Biting oneself deliberately, e.g., on the mouth or lips	28.2	81.65 ± 229.93
8. Deliberately scratching oneself to trigger bleeding	16.6	51.99 ± 286.33
9. Striking oneself with one’s fist or head against a hard object	31.3	79.63 ± 302.97
10. Deliberately scratching one’s own skin	26.6	70.90 ± 256.92

*Number of incidents in the past 12 months

### Descriptive and correlation studies

The associations between NSSI with juvenile BPFs (BPFS-C scores) and dimensions of emotional regulation (ERQ-CA scores) are summarized in Table 2. According to the Pearson’s correlation analysis, NSSI occurrence was negatively correlated with emotional regulation overall (r = -0.159, p < 0.01) but positively correlated with the expression suppression dimension (r = 0.079, p < 0.01), while there was no significant correlation between the cognitive reappraisal dimension and expression suppression (r = 0.022, p > 0.05). The occurrence of NSSI was also positively correlated with BPFs overall (r = 0.314, p < 0.01) and with the dimensions affective instability, identity disorder, and self-harm, but not with the negative interpersonal relationships dimension.

**Table 2:** Correlations between NSSI occurrence with BPFs (BPFS-C scores) and emotional regulation dimensions (ERQ-CA scores) among adolescents with depression

correlation	S.D	1	2	3	4	5	6	7	8	9
1	30.47 ± 6.11	1^**^								
2	16.92 ± 5.06	0.246^**^								
3	13.56 ± 3.38	-0.079^**^	0.022							
4	77.15 ± 17.67	0.159^**^	0.835^**^	0.569^**^						
5	20.11 ± 5.36	-0.314^**^	-0.420^**^	0.229^**^	-0.219^**^	^*^				
6	19.84 ± 5.23	-0.280^**^	-0433^**^	0.135^**^	-0.282^**^	0.891^**^				
7	18.49 ± 4.72	-0.231^**^	-0.270^**^	0.279^**^	-0.069^*^	0.863^**^	0.672^**^			
8	18.71 ± 4.50	-0.195^**^	-0.344^**^	0.188^**^	-0.179^**^	0.831^**^	0.638^**^	0.642^**^		
9	1.61 ± 1.00	-0.379^**^	-0.409^**^	0.194^**^	-0.229^**^	0.883^**^	0.726^**^	0.670^**^	0.630^**^	

(1) NSSI (2) Cognitive reappraisal (3) Expression suppression (4) ERQ-CA (5) Borderline personality features (6) Affective instability (7) Identity disorder (8) Negative relationships (9) Self-harm. Dimensions 2 and 3 belong to the ERQ-CA (4), while dimensions 6, 7, 8, and 9 are part of the BPFS-C (5) (*P < 0.05, **P < 0.01).

The differences in scores between depressed adolescents with and without NSSI are summarized in Table 3. Total and dimension scores on the BPFS-C were significantly higher in the NSSI group than the non-NSSI group. In contrast, the cognitive reappraisal dimension score on the ERQ-CA was higher in the non-NSSI group, while the expression inhibition dimension score was higher in the NSSI group.

**Table 3:** Differences in ERQ-CA and BPFS-C scores between depressed patients with and without NSSI-related behaviors

	NSSI Behaviors No NSSI (n = 342)	NSSI (n = 850)	F	P
Cognitive reappraisal	18.73 ± 4.80	15.98 ± 4.93	76.78	< 0.001
Expressive suppression	13.19 ± 3.78	13.78 ± 3.19	7.51	< 0.001
Borderline personality traits Emotional instability	69.19 ± 17.31 17.98 ± 5.38	81.27 ± 16.25 21.27 ± 4.97	129.98 101.58	< 0.001 < 0.001
Identity disorder Negative interpersonal relationships Self -harm	18.14 ± 5.33 17.13 ± 4.85 15.93 ± 4.44	20.78 ± 4.90 19.15 ± 4.47 20.08 ± 4.63	67.31 47.21 199.66	< 0.001 < 0.001 < 0.001

### Mediating effect of emotional regulation on the association between BPFs and NSSI

**Fig. 1:**
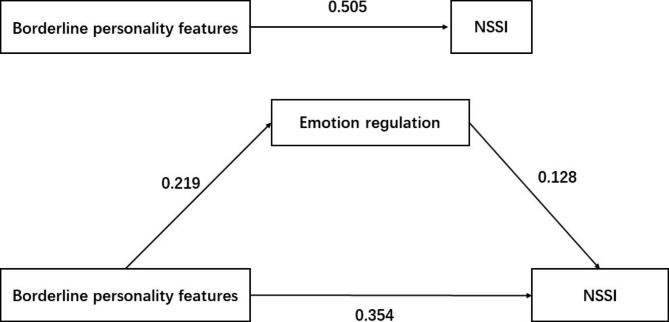
Mediating model of emotional regulation in adolescents with depression

According to the results of the above correlation analysis, the mediation effect was analyzed by using the SPSS macro program PROCESS compiled by Hayes. Under the condition of Model 4, the structural equation was established with BPFs as the independent variable, so as to construct a mediation model based on emotion regulation is constructed (Table 4 ). In Step 1, the regression analysis revealed that the estimated effect size of BPFs on NSSI was 0.505 (P < 0.05). In step 2, the regression coefficient was standardized and the estimated effect size of BPFs on NSSI was 0.354 (P < 0.05). The difference coefficient test indicated that emotional regulation mediated the association between BPFs and NSSI with an effect size of 0.151 ( Fig. 1 ). Thus, BPFs not only directly influenced NSSI behavior, but also indirectly influenced NSSI behavior through emotional regulation.

**Table 4:** Mediating effect of emotional regulation on the association between BPFs and NSSI

		β	SE	estimate	T	P
Emotional regulation	BPFS-C	-0.077	0.010	0.219	7.821	< 0.05
NSSI	Emotional regulation	0.041	0.012	0.128		< 0.05
	BPFS-C	-0.040	0.004	0.354	10.11	< 0.05

## Discussion

Borderline personality features, as measured by BPFS-C scores, were strongly associated with the presence of NSSI behaviors in adolescents with depression. Moreover, poor emotional regulation, measured by the ERQ-CA, was also associated with NSSI behaviors and with BPFs. Further analyses indicated that emotional regulation mediated the association between BPFs and NSSI. Thus, BPFs increased the propensity for NSSI both directly and indirectly via emotional regulation. These findings provide a theoretical basis for the treatment of NSSI in adolescents with depression.

### Characterization of NSSI among depressed adolescents

Bennardi and colleagues found that drug misuse was the most common form of NSSI, followed by scratching [27]. In our study, the majority of this patient cohort (850 of 1192, 71.3%) had demonstrated NSSI behaviors in the previous 12 months, most frequently intentionally cutting or scratching the skin, while Benjet and colleagues reported that the most common NSSI behavior was cutting followed by carving [28]. Further, these discrepancies may be related to cultural differences, stricter control over drug misuse in China [29], and/or to impulsivity having a stronger influence on the studied cohort, which often forces a scratch or cut to be the easiest and most convenient method [30].

### Relationships among NSSI, BPFs, and emotional regulation

Affective disorder is an important factor influencing the onset of NSSI [31]. Studies have shown that individuals prone to mood disorders are more likely to develop NSSI. A review of the existing literature showed that individuals with BPD are more likely than others to develop mood disorders, but it seems that these individuals tend to develop a “strategy” (emotional regulation) to avoid the occurrence of mood disorders. In summary, this study found that NSSI was negatively correlated with emotional regulation. On this basis, we expanded our sample size to explore the relationship between emotional regulation and NSSI, and further examined the association between emotional instability and impulsivity (which is a BPF) and the associations of both of these traits with NSSI behavior. The results showed that emotional regulation was significantly negatively correlated with NSSI, and BPFs were significantly negatively correlated with emotional regulation, suggesting that greater emotional regulation could reduce the risk of BPFs and the occurrence of NSSI behaviors. While both groups did not differ significantly with regards to expressive inhibition scores and cognitive reappraisal scores, several models of adolescent brain development suggest that the maturity gap between cognitive control and emotional processes may explain why adolescents tend to make more emotional decisions, which may lead to an increase in risky behaviors [32]. This apparent conflict could be due to different standard variables ( severity versus history of self-injury ) and possible age confusion, or it could be that the use of expressive inhibition decreased over time in children and adolescents aged 9–15 years, while cognitive reappraisal was stable in this age group [33]. Others have posited that cognitive reappraisal, a core domain of emotional regulation, is two-sided and that negative emotions need to be suppressed or the reaction will cause great damage; thus, expression inhibition is also necessary. This may explain why we found no significant correlation between cognitive reappraisal and expression suppression. Therefore, it is necessary to further study the role of cognitive reappraisal and expression inhibition in NSSI.

Peters and colleagues found that the BPFs, namely impulsivity and emotional instability, were associated with the occurrence of NSSI in subjects without a previous history of NSSI, while in subjects with a history of NSSI, only emotional instability predicted NSSI occurrence. However, the study found that the dimensional model of BPD may be particularly applicable to adolescents, as the dimensional approach may be able to better explain the developmental variability and heterogeneity observed in this age group. Therefore, the current study attempted to analyze BPFs by considering multiple dimensions (which are explained in detail in the research tool); specifically, four dimensions of borderline personality were measured using the BPFS-C, which could be beneficial in examining the impacts of identity disorder, negative interpersonal relationships, and self-harm (lack of impulse control) as well as impulsive emotional instability on NSSI behavior. Indeed, we found that all four dimensions were predictive of NSSI. It is reported that NSSI is inflicted in an effort to avoid interpersonal interactions or tasks in about 15% of patients with BPD and avoidant personality disorder, and to avoid social interaction in about 8% of these patients [34–37]. However, the current study did not explore the association between negative relationships (a borderline personality trait) and NSSI because FASM-C is more likely to reveal recent or current negative evaluations of close friends rather than current poor relationships with teachers or family and all past negative relationships. Nonetheless, studies have also shown that the core functional areas of BPD are affective disorders, separation insecurity, depression, impulsivity, and risk-taking, which are intrinsically linked and may be related to insecure attachment styles [38].

### The mediating role of emotional regulation

According to the classical avoidance model [8], poor emotional regulation is a strong risk factor for NSSI. Such individuals tend to avoid negative emotions through NSSI behavior, thereby temporarily relieving negative emotional affect, but emotional relief further strengthens and promotes the maintenance of NSSI behavior. Hu-Bert and colleagues found that NSSI motivation in female patients with BPD was predictive of NSSI behavior [1], while Anderson and colleagues [39] found that emotional distress promoted emotional dysregulation and avoidance among eating disorder patients. Improper emotional regulation mediates the relationship between emotional distress and avoidance, which in turn is directly related to NSSI behavior. Based on these previous findings, we constructed an affective mediation model and found that BPFs can influence NSSI behavior both directly and indirectly through emotional regulation, a finding that provides important clues for the treatment of NSSI in adolescents with depression. Peters attempted to analyze emotional regulation and NSSI by controlling for dimensional BPD (but not classification-based BPD), and the influence of depression on emotional regulation of NSSI in young participants became insignificant [40]. Thus, mood regulation mediates the relationship between BPFS and NSSI when controlling for current psychological distress (depressive symptoms), but we explored this relationship for further attempts to control BPFs. These results suggested a correlation between BPFs and NSSI with respect to emotional regulation. The findings may be at odds with previous studies that have highlighted the mediating role of depressive symptoms.

## Limitations and prospects

Adolescents have unstable personality characteristics and their capacity for emotional regulation is also different from adults. The current research involved 1,192 patients from 12 hospitals. A multi-faceted and multi-dimensional study was carried out to investigate NSSI style and its association with BPFs and emotional regulation. However, there are also many limitations in our study. First, we only conducted a cross-sectional study, focusing only on adolescents with BPFs and self-injury behaviors in the past 12 months, and did not follow up the enrolled patients. The next step is to follow up the participants to assess adult BPFs, changes in emotional regulation, and the association with NSSI behaviors. Second, there are many theoretical models of emotional regulation. This study only employed the empirical avoidance model. Future research can further explore multiple models of emotional regulation. In addition, the influencing factors of NSSI in adolescents are complex. This study only examined the influence of BPFs and emotional regulation. In addition, the influencing factors of NSSI in adolescents are complex. We only discussed the influence of borderline personality traits and emotional regulation. In order to further explore the symptoms of post-traumatic stress disorder, suicide attempts, suicidal behaviors and comorbidities with other diseases (such as anxiety disorders, eating disorders, etc.), Therefore, our future studies need to further explore the pathogenesis and influencing factors of NSSI in patients with depression, so as to provide theoretical support for finding intervention targets and conducting behavioral intervention research on NSSI in adolescents.

## Conclusion

The results of this study demonstrated that BPFs and poor emotional regulation increased the risk of NSSI among adolescents with depression. The findings highlight the need to investigate the ways in which emotional regulation and BPFs interact to influence NSSI. In addition, future research should broaden the methods used for testing BPFs, abnormal mood regulation, and NSSI behavior in order to examine clinically-relevant research questions, which may in turn help to identify adolescents who are most in need of early intervention.

## Data Availability

The data that support the findings of this study are available from Hefei Fourth People’ Hospital but restrictions apply to the availability of these data, which were used under license for the current study, and so are not publicly available. Data are, however, available from the authors upon reasonable request and with permission of Shenzhen Kangning Hospital. To obtain the data in this study, the researchers may be contacted at yangxi12202022@126.com.
